# Antidepressant Drugs for Chronic Urological Pelvic Pain: An Evidence-Based Review

**DOI:** 10.1155/2009/797031

**Published:** 2010-02-14

**Authors:** Christos Papandreou, Petros Skapinakis, Dimitrios Giannakis, Nikolaos Sofikitis, Venetsanos Mavreas

**Affiliations:** ^1^Postgraduate Program in the Management of Pain, University of Ioannina School of Medicine, Greece; ^2^Department of Psychiatry, University of Ioannina School of Medicine, Greece; ^3^Department of Urology, University of Ioannina School of Medicine, Greece

## Abstract

The use of antidepressant drugs for the management of chronic pelvic pain has been supported in the past. This study aimed to evaluate the available evidence for the efficacy and acceptability of antidepressant drugs in the management of urological chronic pelvic pain. Studies were selected through a comprehensive literature search. We included all types of study designs due to the limited evidence. Studies were classified into levels of evidence according to their design. Ten studies were included with a total of 360 patients. Amitriptyline, sertraline, duloxetine, nortriptyline, and citalopram are the antidepressants that have been reported in the literature. Only four randomized controlled trials (RCTs) were identified (two for amitriptyline and two for sertraline) with mixed results. We conclude that the use of antidepressants for the management of chronic urological pelvic pain is not adequately supported by methodologically sound RCTs. From the existing studies amitriptyline may be effective in interstitial cystitis but publication bias should be considered as an alternative explanation. All drugs were generally well tolerated with no serious events reported.

## 1. Introduction

Chronic pelvic pain is a complex, poorly understood health problem with prevalence rates ranging from 2.7% to 5.7% [1, 2].

The European Association of Urology defines chronic pelvic pain as a nonmalignant pain, perceived in structures related to the pelvis of either men or women and constant or recurring over a period of ≥6 months. In all cases there are often, associated with negative cognitive, behavioural and social consequences [3].

Chronic pelvic pain is better viewed as a functional syndrome involving multiple sites, aetiologies and mechanisms. The International Continence Society has defined this syndrome as persistent or recurrent episodic pelvic pain associated with symptoms suggesting lower urinary tract, sexual, bowel, or gynaecological dysfunction, without evidence of infection or other obvious pathology [4]. Therefore, concerning the urological system it can be bladder pain syndrome/interstitial cystitis, urethral pain syndrome, prostate pain syndrome, scrotal pain, or penile pain syndrome [3].

The analgesic properties of antidepressant drugs were first reported 40 years ago, and they are now widely used for the treatment of chronic and neuropathic pain. Their efficacy has now been shown in randomized controlled trials (RCTs), systematic reviews, and meta-analyses [5, 6]. It is also worth noting that their analgesic effect seems to be independent of their antidepressant effect [6]. Several controlled studies support the efficacy of antidepressants in different chronic pain conditions, but little is known about their efficacy in chronic pelvic pain [7]. This systematic review aims to assess the available evidence for the efficacy and acceptability of antidepressant drugs in chronic urological pelvic pain and to provide data necessary to design future trials. 

## 2. Methods

### 2.1. Search Strategy

We searched PubMed and Embase for studies in English language published from 1966 to December 2008. We used the following search string: (antidepressants OR amitriptyline OR paroxetine OR fluoxetine OR duloxetine OR venlafaxine OR imipramine OR desipramine OR sertraline OR citalopram OR doxepin) AND (chronic pelvic pain OR interstitial cystitis OR prostatitis OR penile pain OR urethral pain OR scrotal pain). Additional strategy for identifying trials included searching the reference list of the studies included.

### 2.2. Inclusion and Exclusion Criteria

We included all randomized-controlled trials, nonrandomized controlled trials, uncontrolled trials (prospective case series), and observational studies that retrospectively reviewed medical charts (retrospective case series). All reviews and studies in which the pharmacological intervention did not include any antidepressant agent or included combination of agents were excluded. The decision of including nonrandomized and uncontrolled studies was based on the limited number of controlled trials available. Safety and acceptability issues are also important in this group of patients. For efficacy analysis data on chronic pelvic pain management, intensity and duration of pain were considered. Other outcomes measured by the authors were also considered.

### 2.3. Data Extraction and Assessment of Methodological Quality

Data extraction was performed independently by two of the authors (CP and PS) and checked by another (DG). In case of disagreement two senior authors (PS and DG) reviewed the studies and reached a consensus. We used the Jadad scale [8] to assess the methodological quality of all included randomized controlled trials. We did not assess the methodological quality of uncontrolled trials or retrospective reviews as the evidence resulting from these types of study is relatively weak and uncertain.

### 2.4. Classification of Study Designs

Firstly we classified the retrieved papers into either intervention or nonintervention studies. 

In intervention studies the investigators intentionally change some aspect of the status of the subject, for example by introducing a new therapeutic regimen. We further classified intervention studies into the following categories:

- randomized controlled trial (random allocation of eligible and consenting patients into groups to receive or not to receive the study intervention),
- nonrandomized controlled trial (similar to the randomized but without random allocation of patients to the study and control groups),
- consecutive case-series (a prospective clinical study that includes all eligible patients identified by the researchers during the study registration period. The patients are treated in the order in which they are identified. This type of study usually does not have a control group. A synonym of this study is “open-label” uncontrolled trial and prospective case-series),
- nonconsecutive case series (a prospective clinical study that includes some, but not all, of the eligible patients identified by the researchers during the study registration period. This type of study does not usually have a control group. These studies are also referred to the literature as prospective case-series),
- nonintervention studies in the context of the present review were considered all studies that retrospectively reviewed medical charts of patients. In these studies the investigators did not see or examined the patients but systematically extracted patient data from the medical charts.

For the definition of consecutive and nonconsecutive case series we used the terminology used in the US. National Cancer Institute Dictionary of terms (http://www.cancer.gov/dictionary/).

### 2.5. Levels of Evidence

Based on the study design we categorized the level of evidence that each paper represented following the guidelines developed by the Agency for Healthcare Research and Quality [9, 10]. We defined five levels of evidence:

- Level I: Meta-analysis of multiple well-designed randomized controlled trials (since we could not identify any meta-analysis of randomized controlled trials related to the study aims, this level was not assessed in this review),

(ii) Level II: Randomized Controlled trial,

(iii) Level III: Nonrandomized controlled trial (IIIA), uncontrolled trials, consecutive case series, and nonconsecutive case series (IIIB),

(iv) Level IV: Retrospective case series based on systematic investigation of medical charts,

(v) Level V: case reports, clinical examples, expert opinions, and so forth, (this type of evidence was not assessed in this review).

Safety profiles and tolerability of the antidepressants considered are described using a narrative, qualitative, approach. Adverse effects reported in all trials analyzed in this review are described. 

## 3. Results

**Figure 1:**
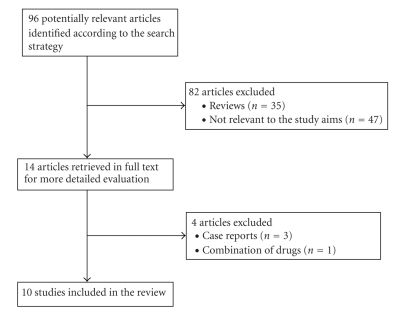
Flow Diagram of the study.

The results of our search strategy are presented in Figure 1. Ten studies were included in this review and their characteristics are summarized in Table 1 [11–20]. Of these studies, 4 were RCTs, 4 were consecutive (prospective) case series, 1 non consecutive (prospective) case series and 1 retrospective case series. Five studies investigated the efficacy of amitriptyline and two studies investigated sertraline. Of the remaining three studies, 1 investigated nortriptyline, 1 duloxetine, and 1 citalopram.

### 3.1. Amitriptyline

Van Ophoven et al. [11] conducted an RCT in 50 patients with interstitial cystitis (IC), who were randomly assigned to amitriptyline or placebo. Patients were prospectively treated for 4 months with a self-titration protocol that allowed them to escalate drug dosage in 25 mg increments in 1-week intervals (maximum dosage 100 mg). The primary outcome measure was the change from baseline in the O'Leary-Sant Interstitial Cystitis Symptom Index (ICSI). Changes in intensity of pain and frequency were chosen as secondary outcome parameters. The study was of high quality according to the Jadad scale (score: 5). Amitriptyline treatment was associated with a mean reduction in symptom score of 8.4 compared to 3.5 for the placebo group. The difference was statistically significant (*P *= .005) and perhaps clinically significant. Pain and urgency intensity also improved significantly in the amitriptyline group compared to the placebo group (*P *< .001). Anticholinergic side effects were reported by all except 2 patients in the amitriptyline group (92%) and by 5 patients in the placebo group (21%). The side effects in the amitriptyline group that occurred at a greater incidence than in the placebo group were mouth dryness (79% versus 8%), weight gain (63% versus 8%), sedation (37.5% versus 12.5%), constipation (45.8% versus 8%), nausea (12.5% versus 0%), blurred vision/diplopia (17 % versus 0%), and erectile dysfunction (4% versus 0%). 

Sator-Katzenschlager et al. [12] compared the efficacy and acceptability of amitriptyline, gabapentin and their combination in 56 women with chronic pelvic pain in an open-label, randomized, controlled trial. The primary outcome measure was the change from baseline in pain intensity. This study had a low Jadad quality score of 2, since it was unblinded and the authors did not describe the method they used for the random allocation of subjects. Doses of amitriptyline and gabapentin were increased to maximum daily doses of 150 mg and 3600 mg, respectively, until sufficient pain relief or the occurrence of side effects. All patients experienced significant pain relief during the observation period (*P *< .0001). However, monotherapy with amitriptyline achieved less pain relief compared to the other two treatment approaches. There was no significant difference between the groups in the incidence of severe side effects, requiring discontinuation of treatment.

Van Ophoven and Hertle [13] evaluated amitriptyline's safety and efficacy in interstitial cystitis in a consecutive (prospective) case series that included 94 patients. Amitriptyline was received following an established self-titration protocol without a limitation of the maximum daily dose. Mean treatment duration was 16.5 ± 7.2 months. The primary outcome measure was response defined by the Global Response Assessment questionnaire. Further efficacy measures included changes in pain and urgency. Overall response to treatment was observed in 60 patients (64%). Pain and urgency intensity were improved significantly compared with baseline (*P *< .05). Side effects occurred in 79 patients (84%). They contributed to the decision to drop out in 25 of the 29 patients (86%) and they were the primary reason for drop out despite an initial treatment response in 9. Dry mouth was the most frequent side effect.

Hanno et al. [14] treated 25 patients with interstitial cystitis in nonconsecutive case series. The initial amitriptyline dose of 25 mg was increased gradually during a 3-week period to 75 mg. The authors evaluated the efficacy of treatment in reducing pain, daytime urinary frequency, nocturia. A significant improvement in pain and daytime frequency was reported (*P *< .05). Nocturia did not improve significantly (*P *= .235). The adverse effects reported were drowsiness, dry mouth, and weight gain.

Pranikoff and Constantino [15] reviewed the charts of 22 patients with urinary frequency or genital, pelvic, or suprabubic pain syndromes. All were treated with amitriptyline in doses ranging from 25 to 100 mg. Eleven patients became symptom-free, six showed significant improvement, and five did not respond. Of the nonresponders, four could not tolerate the medication and one patient did not see any symptom improvement.

### 3.2. Sertraline

Engel et al. [16] conducted an RCT in order to assess the efficacy of sertraline in 23 women suffering from chronic pelvic pain. Random assignment was to either 50 mg of sertraline taken twice daily or to the same regimen of an inactive matching placebo. After 6 weeks, both groups were switched to single-blind placebo for 2-weeks. After the washout, subjects on placebo during the first study stage were crossed-over to sertraline for a second 6-week stage, while those on sertraline were crossed-over to placebo. The primary outcome of interest was pelvic pain intensity estimated by a Composite Pain Intensity score. This study had a Jadad quality score of 4, since the authors did not report the random allocation procedure. There were no significant improvements in pain noted on sertraline compared to placebo. The authors did not provide information about side effects.

Lee et al. [17] conducted a small RCT in 14 men with chronic pelvic pain syndrome. Patients were randomized to receive in a blind double fashion sertraline 50 mg daily or matched placebo for 13 weeks. After that period both investigators and patients were unblinded. Subjects initially randomized to receive sertraline were given the option to continue this for a further 13 weeks and those receiving placebo were given the opportunity to cross-over into sertraline. Prostatic symptom frequency (PSF) and severity (PSS) scores were completed. The study had a Jadad quality score of 2, since it was unblinded and the authors did not report the random allocation procedure. There was no statistically significant difference between the two groups. The authors noticed a trend towards sertraline being associated with an improvement in PSF and PSS scores. The authors mentioned that sertraline was well tolerated, with no further information provided about side effects.

### 3.3. Nortriptyline

Walker et al. [18] reported findings from a consecutive (prospective) case series. A total of 14 women with chronic pelvic pain were treated with nortriptyline 100 mg per day after a 2 week upward titration. The primary end point was the reduction in pain. At the two-month evaluation six of the seven women who remained in treatment were either pain free or reported that their pain was significantly better. The rest 8 women had been dropped out. The study did not include information on side effects.

### 3.4. Duloxetine

Van Ophoven and Hertle [19] conducted a consecutive (prospective) case series in order to evaluate the efficacy and tolerability of duloxetine for interstitial cystitis. A total of 48 women were treated for 2 months following an uptitration protocol to the target dose of 40 mg taken twice daily. The primary end point was a change in the overall well-being evaluated by a patient reported Global Response Assessment. Secondary outcome measures were changes in pain and urgency. Only 5 responders out of 48 patients (10.8%) were identified. Regarding secondary end points, duloxetine treatment did not result in statistically significant improvement of pain and urgency. The tolerability of the drug was poor, mainly due to nausea. A total of 17 patients (35.4%) dropped out of the study exclusively due to side effects.

### 3.5. Citalopram

Brown et al. [20] evaluated citalopram's efficacy in the treatment of chronic pelvic pain in a consecutive (prospective) case series that included 14 patients. Citalopram dosage ranged from 20 to 60 mg per day. The primary outcome measure was change in pain severity. The authors also assessed the functional disability response to citalopram. Pain severity showed a trend towards improvement on the McGill Intensity Scale (*P* = .096), but there was no significant differences on the pain disability Index (*P* = .158). The most commonly adverse effects reported were headache, dry mouth and abdominal pain.

## 4. Discussion

The main finding of this review is that the use of antidepressants in the management of chronic urological pelvic pain is not supported by an adequate number of well designed randomized controlled trials.

Amitriptyline and sertraline are the drugs that have been studied more extensively in chronic urological pelvic pain. Regarding the first, we identified two RCTs that randomized 106 patients recruited [11, 12] and two prospective case series with a total of 119 patients [13, 14]. Regarding sertraline, we identified two RCTs with a total of 37 patients [16, 17]. According to this review most of the studies conducted so far are uncontrolled prospective case series. 

Van Ophoven's study [11] with amitriptyline for interstitial cystitis was a high-quality double blind RCT with a small percentage of dropouts and a considerable number of patients. Engel's study [16] with sertraline for chronic pelvic pain provides good level of evidence, but it is of lower validity. Lee's study [17] with sertraline is the only one which was conducted for chronic prostatitis, but it has low validity and methodological deficiencies. Sator-Katzenschlager's RCT [12] comparing amitriptyline with gabapentin did not include placebo and therefore it is difficult to evaluate efficacy for amitriptyline.

For nortriptyline, duloxetine, and citalopram only uncontrolled studies were identified [18–20]. These studies have the lowest level of evidence and no firm conclusions for the efficacy of these antidepressants agents can be extracted. Therefore, their use should still be considered as experimental. 

Amitriptyline was found to be effective compared to placebo in interstitial cystitis. However, we could only identify a single RCT and therefore it is very likely that publication bias could offer an alternative explanation. 

Regarding acceptability, we found that antidepressants were generally safe drugs with tolerable side effects. The withdrawal rates in most studies were not high and the reported reason for the withdrawal was not relevant to the side effects. Nonetheless, most of the studies were of short-term duration and it is not known whether acceptability would be the same on the long-term. Two amitriptyline studies were of longer duration [12, 13] and have shown that long-term use may be well tolerated. 

A major problem in evaluating the efficacy of antidepressants in chronic urological pelvic pain is the heterogeneity of the studies concerning the classification of chronic pelvic pain syndrome. In this review we focused on studies examining chronic urological pelvic pain. However, some of the included studies consisted of mixed population in terms of pain location, such as patients with pain perceived in gynaecological system. 

Antidepressants may be effective in chronic pelvic pain either by acting directly on the neural mechanisms of pain or by reducing depressive symptoms that may influence the experience of pain or the capacity to cope with the pain. Regarding the first, studies have shown that the spinal and neuronal pathways are modulated by activation and/or inhibition of neurons in the periphery, at spinal levels and at supraspinal regulatory sites. Serotonergic pathways and receptor mechanisms play a crucial role within this neuronal network. The antidepressants alleviate symptoms probably via mechanisms such as blockade of acetylcholine receptors, inhibition of reuptake of released serotonin and norepinephrine, and blockade of the histamine H1 receptor [21].

Regarding the role of psychological morbidity, epidemiological studies of chronic pain have shown the strong association between depressive symptoms and experience of pain in general [22] and chronic pelvic pain in particular [23]. Banks and Kerns have proposed a diathesis-stress framework to explain the nature of this relationship. According to this model individuals with a higher “diathesis” for depression (biologically or genetically determined) are more likely to develop an actual episode of depression in the presence of a stressor such as chronic pain. These authors suggest that the treatment of chronic pain can be improved if depression is considered an essential part of the patient's clinical presentation [24]. This view may have practical clinical implications since, even if there is little evidence from randomized controlled trials to routinely recommend the use of antidepressants in all patients with chronic pelvic pain syndrome, the presence of comorbid psychological dimension may increase the likelihood of a positive outcome. 

The best way of studying the effect of antidepressants is by using carefully designed placebo-controlled double blind studies. More RCTs of longer duration with larger numbers of patients suffering from chronic urological pelvic pain are needed. Future studies should include patient subgroups on the basis of symptom severity or predominant symptom. It may be advisable to stratify the sample with respect to the presence or absence of major depression to further evaluate the efficacy of antidepressants. Studies should also include measures of disability and coping. Combination or multimodal therapy with antidepressants and other potentially beneficial agents (e.g., anti-inflammatory agents, a-blockers) that have independent actions should be evaluated in RCTs. Because a given pharmacologic intervention might selectively alleviate a symptom while causing adverse effects or exacerbating other symptoms, future trials should assess both side effects and overall health-related quality of life by using validated measures.

## 5. Conclusions

Antidepressants are safe generally tolerable drugs and may have a place in the treatment of chronic urological pelvic pain. Amitriptyline, in particular, may be useful in the management of interstitial cystitis, but the effect of publication bias cannot be estimated. For this reason our main conclusion is that we need further research with adequately designed RCTs in order to better evaluate the exact role of antidepressants on chronic urological pelvic pain.

## Figures and Tables

**Table 1:** Characteristics of studies assessing the efficacy of antidepressants in chronic pelvic pain.

Study	Study	Level of	Diagnosis	Antidepressants—	Control group	Sample size	Duratin of	Main outcome	Results
	design^a^	evidence^a^		dosage		(age rang)	study	Measures	
Van Ophoven et al. 2004 [11]	RCT	II	Interstitial cystitis	Amitriptyline—25–100 mg/day	placebo	*N* = 50 (NA)	4 months	Change from baseline in the O'Leary-Sant Interstitial Cystitis Symptom Index (ICSI)	Mean reduction in symptom score of 8.4 for amitriptyline versus 3.5 for the placebo group (*P* < .005)
Sator-Katzenschlager et al. 2005 [12]	RCT	II	Chronic pelvic pain	Amitriptyline—25–150 mg/day	Gabapentin/Gabapentin + Amitriptyline	*N* = 56	24 months	Change from baseline in pain intensity	Significant pain relief in all patients. Pain relief significantly better in patients receiving gabapentin either alone or in combination with amitriptyline than in patients on amitriptyline alone
Van Ophoven and Hertle 2005 [13]	Consecutive (prospective) case series	IIIB	Interstitial cystitis	Amitriptyline—12.5–150 mg/day	uncontrolled	*N* = 94 (NA)	mean:19 month sd:12.5	Change from baseline in the overall well-being (global response assessment questionnaire)	Statistically significant improvement in symptoms compared to baseline
Hanno et al. 1989 [14]	NonConsecutive (prospective) case series	IIIB	Interstitial cystitis	Amitriptyline—75 mg/day	uncontrolled	*N* = 25 (21–83)	mean:16.4 months (4-40)	Change from baseline in pain, daytime frequency, nocturia	Statistically significant improvement in pain and daytime frequency compared to baseline. No significant improvement in nocturia
Pranikoff and Constantino 1998 [15]	Retrospective case series	IV	Chronic urinary frequency and pain	Amitriptyline—25–100 mg/day	uncontrolled	*N* = 22 (21–76)	3 months	Change from baseline in symptoms	Complete resolution of symptoms in 11 patients and some residual symptoms present in 6.
Engel et al. 1998 [16]	RCT	II	Chronic pelvic pain	Sertraline—100 mg/day	placebo	*N* = 23 (19–45)	14 weeks	Change from baseline in the Composite Pain Intensity score	No significant improvement in pain compared to placebo
Lee et al. 2005 [17]	RCT	II	Chronic pelvic pain	Sertraline—50 mg/day	placebo	*N* = 14 (18–65)	26 weeks	Change from baseline in prostatic symptom frequency and severity scores	No significant improvement in pain severity (*P* = .34) and a nonsignificant trend for improvement in pain frequency (*P* = .09) compared to placebo
Walker et al. 1991 [18]	Consecutive (prospective) case series	IIIB	Chronic pelvic pain	Nortriptyline—100 mg/day	uncontrolled	*N* = 14 (NA)	1 year	Change from baseline in pain intensity	Significant improvement in pain intensity compared to baseline (*P* < .05)
Van Ophoven and Hertle 2007 [19]	Consecutive (prospective) case series	IIIB	Interstitial cystitis	Duloxetine—80 mg/day	uncontrolled	*N* = 48 (25–69)	2 months	Change from baseline in the overall well-being (global response assessment questionnaire)	No significant improvement in symptoms compared to placebo
Brown et al. 2008 [20]	Consecutive (prospective) case series	IIIB	Chronic pelvic pain	Citalopram— 20–60 mg/day	uncontrolled	*N* = 14 (18–50)	3 months	Change from baseline in pain quality and intensity	No significant differences in the quality of pain. Trend towards improvement in pain intensity (*P* = .096)

RCT: randomized controlled trial; NA: not available; ^a^see methods for definition of study design classification and level of evidence.
